# Altered pattern separation in Goto-Kakizaki rats

**DOI:** 10.1016/j.crneur.2023.100082

**Published:** 2023-03-17

**Authors:** Chelsey C. Damphousse, Jaclyn K. Medeiros, Nicole E. Micks, Diano F. Marrone

**Affiliations:** Dept. of Psychology, Wilfrid Laurier University, Waterloo, ON, N2L3C5, Canada

**Keywords:** Memory, Diabetes, Hippocampus, Pattern separation, Granule cell

## Abstract

Type 2 diabetes mellitus has steadily increased in prevalence over the past five decades. Among the health risks associated with this disorder are cognitive decline and are increased risk of developing dementia. To further investigate the link between diabetes and cognition, here we test memory performance and hippocampal function in the Goto-Kakizaki (GK) rat, a robust model of diabetes. Relative to age-matched Wistar rats, GK rats show impairments in a conjunctive memory task that requires discriminating objects not only on the basis of their physical characteristics, but also on the basis of where and when they were last seen. Concomitant to these deficits are changes in the pattern of expression of *Egr1* (an immediate-early gene critical for memory) in dentate gyrus granule cells, consistent with dentate hypoactivity leading to unstable hippocampal representations. These data support the hypothesis that diabetes confers a phenotype of accelerated senescence on the hippocampus, and help to link this disorder with changes in hippocampal circuits.

## Highlights

• Using Goto-kakizaki (GK) rats, we assess how diabetes alters hippocampal function.
• GKs are impaired at discriminating object/location, but not object/context pairs.
• GKs express Egr1 less consistently in granule cells when stimuli are unchanged.
• This Egr1 expression pattern is consistent with impaired pattern separation in GKs.
• These data inform how diabetes alters information processing within the hippocampus.

## Graphical abstract

**Figure:**
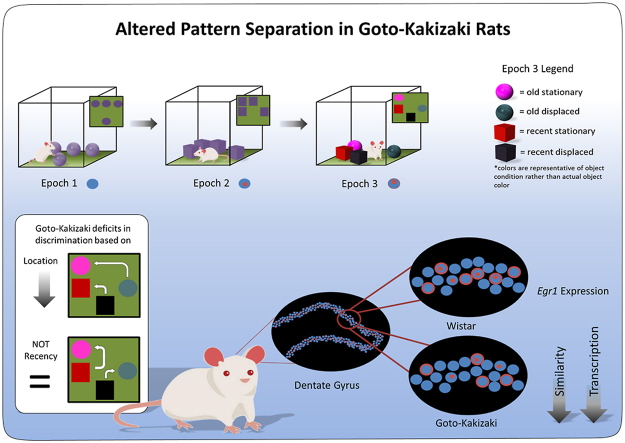

## 1 Introduction

Type II Diabetes mellitus (T2DM) is a common metabolic disorder that has steadily increased in prevalence over the past several decades (Menke et al., 2015). Although eradicating T2DM remains the ideal, treating its complications remains critical for millions of individuals currently afflicted with the disease. Among these complications, T2DM is associated with cognitive decline and increased risk of dementia (e.g., Biessels et al., 2006; Lu et al., 2009; Cukierman-Yaffe, 2014; Palta et al., 2014; Moheet et al., 2015; Chatterjee et al., 2016).

The link between T2DM and cognitive decline has been appreciated for a century (Miles and Root, 1922), and a wealth of studies over this time have investigated diabetes-associated cognitive decline (DACD). A number of brain regions synthesize receptors for insulin, and insulin-like peptides such as insulin-like growth factor-I (IGF-I), glucagon-like peptide-1 (GLP-1), and ghrelin, have significant impacts on the structural and functional plasticity of a number of brain regions (reviewed in Messier and Teutenberg, 2005; Mainardi et al., 2015). Moreover, evidence suggests that these effects may be particularly profound for the hippocampus, a region critical to memory function that contains a high density of insulin receptors (Havrankova et al., 1979; Zahniser et al., 1984; Marks and Eastman, 1990) and where learning has been shown to dynamically regulate insulin receptor function (Zhao et al., 1999). Clarifying how these changes in signalling pathways manifest in changes in information processing in defined neural circuits may be aided through the use of animal models such as the Goto-Kakizaki (GK) rat (Goto et al., 1975; Kimura et al., 1982), which is selectively bred for chronic hyperglycemia and insulin resistance (CHAIR) in the absence of obesity. Like people afflicted with T2DM (Palta et al., 2014; Cukierman-Yaffe, 2014), these rodents display cognitive deficits in a number of tasks that depend upon the hippocampus (e.g., Matsunaga et al., 2016; Tian et al., 2018; Duarte et al., 2019).

It has been proposed that CHAIR undermines cognition in part by accelerating the processes associated with normal aging (Kent, 1976; Lu et al., 2009). Here we test whether this hypothesis extends to information processing in hippocampal circuits. Senescent rats have a number of phenotypical changes in the hippocampus in terms of both behavioural deficits and patterns of activity in hippocampal neuronal circuits (Small et al., 2011; Lester et al., 2017). Searching for these phenotypes in GK rats may inform whether CHAIR also confers accelerated senescence of information processing circuits and may provide novel targets for reducing the impact of this disease.

**Fig. 1:**
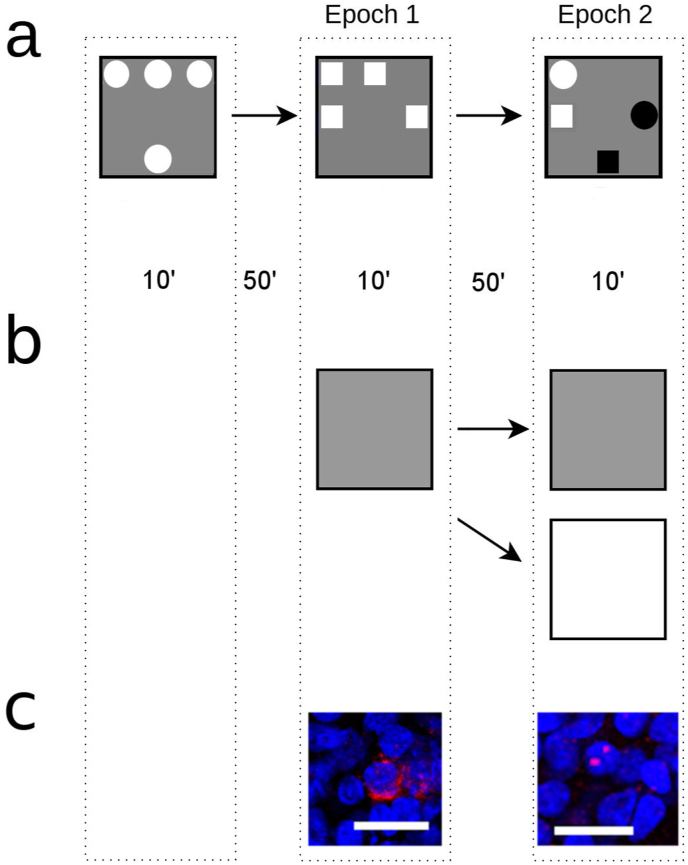
Overview of Experimental Procedures. A schematic (above) demonstrates the placement of objects and timing of trials in (a) the conjunctive memory task (CMT) and (b) environmental exploration (EE), and depicts the relationship between activity during each of these periods and (c) examples of the pattern of Egr1 expression expected (DAPI in blue, Egr1 in red, scale bar = 50 μm). In CMT, rats in the received their first sample trial (left) in an open-field containing 4 identical copies of a novel object (circles) for 10 min. Rats in EE remained in their home cage at that time. As this experience will be 2 h before the animal is sacrificed, Egr1 transcribed at this time will be translated and degraded. After a delay of 50 min, rats in CMT (a) received a second sample trial (middle, Epoch 1) in which four copies of a different object (squares) were present. Two of these objects were placed in locations that already contained objects during sample trial one, while the remaining two objects were randomly placed in novel locations. Rats in EE (b) explored a novel environment for 10 min using established procedures (Guzowski et al., 1999; Marrone et al., 2011). *Egr1* transcribed during this time will have time migrate outside the nucleus for translation, and so will be detected at the time of sacrifice of the animal as a diffuse signal in the surrounding cytoplasm (bottom middle). After an additional delay of 50 min, the rats in CMT (a) received a test trial (right) in which the arena contains two “old” object from sample trial 1 (circles) and two “recent” objects from sample trial 2 (squares). One object of each type was presented in a location in which it had already been encountered (white), while the other object of the same type was in a location where it had never been encountered (black). This combination provides the opportunity to quantify if rats exhibit a preference based on recency or spatial displacement. Rats in EE (b) explored either the same environment (grey) or a different enclosure in a different room (white) for 10 min *Egr1* transcribed during this time will have time will be detected at sacrifice as a punctate intra-nuclear signal (bottom right). (For interpretation of the references to colour in this figure legend, the reader is referred to the Web version of this article.)

The dentate gyrus (DG) is a brain region subject to major phenotypical age-related change in hippocampal circuitry (Small et al., 2011; Schmidt et al., 2012). The DG of aged rodents shows reduced input from the perforant path, and a subsequent rearrangement of normal synaptic inputs and altered dentate granule cell (GC) activity (Geinisman et al., 1992; Barnes and McNaughton, 1980; Nicholson et al., 2004; Marrone et al., 2011). These changes, in turn, are thought to drive hyperactivity in hippocmapal CA3 (reviewed in Leal and Yassa, 2015) and vulnerability to memory interference in aged people and animals by impairing the ability for the hippocampal tri-synaptic circuit to engage in pattern separation. Pattern separation is a process by which neural networks create uncorrelated representations of two or more events despite these events highly correlated sensory input (e.g., from similar visual cues) in order to combat interference and facilitate the selective retrieval of individual episodes of experience (reviewed by Knierim and Neunuebel, 2016). Here we test the hypothesis that senescent activity patterns should be apparent in young GK rats by testing memory performance and *Egr1* (also called *zif268, krox24*) expression in the hippocampus of GK rats and age-matched Wistar rats. Initial testing began with a spatial navigation assay that induces robust *Egr1* expression (Guzowski et al., 1999; Gheidi et al., 2013, see Fig. 1) and that is sensitive to deficits in pattern separation (Marrone et al., 2011). We then tested rats in a conjunctive memory task (CMT) incorporating memory for objects, their location, and the surrounding context (Dere et al., 2005a,b; Kart-teke et al., 2006). The high potential for interference is likely to heavily tax hippocampal pattern separation and provide an optimal test for senescence-like changes in DG information processing under conditions of CHAIR.

## 2 Materials and methods

### 2.1 Subjects

A total of 32 adult (approximately 6 month-old) male GK rats, bred at Wilfrid Laurier University from stock originally obtained from Charles River Canada (St. Constance, QC) and 30 age-matched Wistar Kyoto rats (Charles River Canada, St. Constance, QC) were used in this experiment. All animals were housed individually with *ad libitum* access to food and water on a 12:12-h reverse light cycle. All procedures were approved by the Wilfrid Laurier University Animal Care Council according to the guidelines outlined by the Canadian Council on Animal Care. Subjects engaged in one of 2 tasks: (a) CMT (Dere et al., 2005a,b; Kart-teke et al., 2006), or (b) environmental exploration (Guzowski et al., 1999). Each of these is described below. An additional caged control group (n = 6 rats per strain) was sacrificed directly from the home cage as a negative control.

### 2.2 Conjunctive memory task

To probe cognition and behaviourally-induced *Egr1* expression under conditions with a high potential for memory interference, CMT was conducted (Fig. 1a) following established procedures (Dere et al., 2005a,b; Kart-teke et al., 2006). All testing was conducted in a square open-field with opaque walls measuring 75 cm long and 30 cm high. A web camera connected to a computer running Any-maze (Stoelting, Wood Dale, IL) was used to record and analyze trials. Rats initially received habituation trials in which they were placed in the empty apparatus and allowed to explore freely for 10 min on 3 consecutive days.

One day after the last habituation session, each rat (n = 10 GK and 8 Wistar) received two sample trials and a test trial. On the first sample trial, rats were placed into the centre of the open-field containing 4 identical copies of a novel object placed in the four locations, and allowed to explore them for 10 min. After a delay of 50 min, the rats received a second sample trial identical to the first, except that four novel objects were present. Two of these objects were placed in locations previously occupied during sample trial one, while the remaining two objects were randomly placed in novel locations in which the rat had never seen an object. After an additional delay of 50 min, the rats received a 10 min test trial identical to the sample trials, except that two copies of the object from sample trial 1 (old objects) and two copies of the object known from sample trial 2 (recent objects) were present. One object of each type was presented in a location in which it was already encountered during a sample trial (stationary), while the other object of the same type was presented in a location where it had never been encountered (displaced). Thus, one old object was kept in place (old stationary – OS), while the other was displaced to a novel location (old displaced – OD). The same was done for the recent objects, yielding a recent stationary (RS) object and a recent displaced (RD) object. This combination provides the opportunity to know whether the exploration pattern exhibited by the rats would indicate an interaction between recency and spatial displacement. All objects used were junk objects described previously (Marrone et al., 2018). After each trial, the objects and the open field were thoroughly cleaned with an 0.5% acetic acid solution to remove odour cues.

### 2.3 Environmental exploration

The procedure for the spatial exploration trials is similar to that previously described (Guzowski et al., 1999; Marrone et al., 2011). Briefly, rats were shuttled to a dimly lit room containing several distinct landmarks, and placed within a 61 × 61 cm open field with opaque walls measuring 30 cm high divided into 9 equal grids. During exploration, rats were randomly placed within a different grid every 15 s until each grid was visited. This passive exploration procedure ensures that all aspects of the environment were sampled equally, and has been shown to equalize the induction of activity-dependent genes across groups of animals (Small et al., 2011). Rats were then shuttled back to their colony room cages. The exploration treatment was repeated 50 min later either in the same apparatus (same, n = 8 rats per strain), or to a different open field of the same dimensions located in another room and containing distinct local and distal cues (different, n = 8 rats per strain).

### 2.4 Tissue processing

Rats were anaesthetized immediately after either their final trial in the CMT or their second exploration session and then decapitated. The brains were removed and flash frozen in a container filled with 2-methyl butane that is cooled by a secondary container filled with dry ice immersed in ethanol. The right hemispheres of the brains were mounted in blocks of 8 using OCT compound (Fischer Scientific, Toronto, ON) with all groups represented within each block, including control subjects. Coronal sections of 20 μm were sliced using a Leica CM3050 cryostat (Leica Microsystems, Concord, ON) and thaw mounted onto Superfrost Plus microscope slides (VWR, Toronto, ON). Slides were stored at −80 °C until further processing.

Compartmental analysis of temporal activity by fluorescence *in situ h*ybridization (catFISH) was then conducted as previously described (Guzowski et al., 1999; Gheidi et al., 2013; Grella et al., 2019), digoxigenin-conjugated full-length *Egr1* riboprobes were synthesized using a commercial transcription kit. Slides were thawed, fixed in 4% formaldehyde, bathed in acetic anhydride, methanol:acetone (1:1), and treated with prehybridization buffer at room temperature for 60 min. The slides were then incubated with 100 ng of *Egr1* probe overnight at 56 °C in a humid chamber. Slides were bathed in a series of saline-sodium citrate (SSC) washes, placed in RNase A at 37 °C, bathed in H_2_O_2_, blocked with 0.05% normal sheep serum, and incubated with anti-digoxigenin-horseradish peroxidase antibody for 2 h at room temperature. Slides were labelled with CY3 (Fluorescent Solutions, Augusta, GA) and counterstained with 4′-6-diamidino-2-phenylindole (DAPI). Coverslips were applied with antifade medium and sealed. All reagents are from Sigma-Aldrich Canada (Oakville, ON) unless otherwise specified.

### 2.5 Imaging

**Fig. 2:**
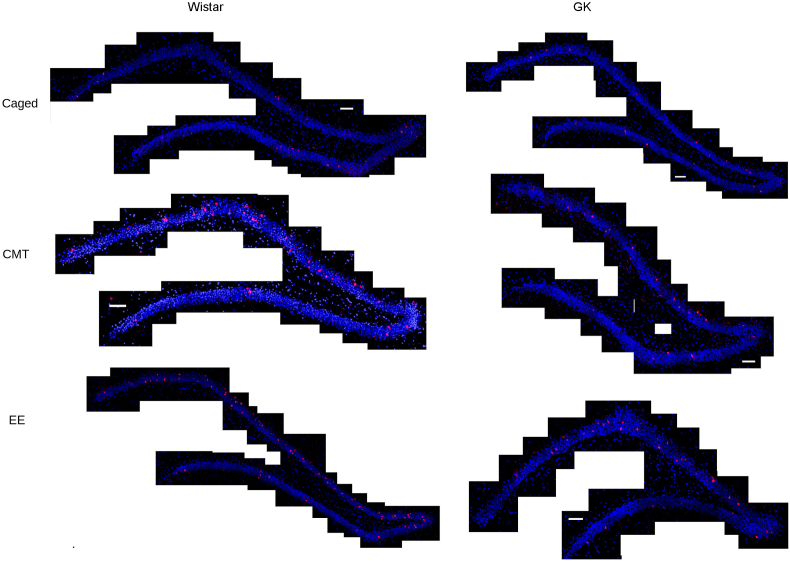
Task-dependent *Egr1* transcription. Sample tile-scans (scale bar = 100 μm) showing *Egr1* (red) transcription in DPI-stained (blue) of the dorsal dentate gyrus from each group show that caged control (caged) animals show very little basal Egr1 transcription. This is true of both Wistar (left) and Goto-Kakizaki (GK, right) rats. A robust up-regulation of *Egr1* transcription can be seen, largely in the suprapyramidal blade, in rats that engaged in the conjunctive memory task (CMT, middle) or environmental exploration (EE, bottom). (For interpretation of the references to colour in this figure legend, the reader is referred to the Web version of this article.)

Using an Olympus FV1000 confocal microscope (Olympus Canada, Mississauga, Ontario Canada), 20 μm thick z-stacks (optical thickness: 1.1 μm, interval: 0.7 μm) were obtained from the suprapyramidal blade of the DG (Fig. 2), which is also referred to as the inner/enclosed blade (Witter, 2007). This region of the DG was chosen for analysis because more IEG expression is consistently observed in the supra-pyramidal blade during spatial tasks. In fact, the infrapyramidal blade of the DG is typically nearly silent under these conditions (Temple et al., 2003; Chawla et al., 2005; Ramirez-Amaya et al., 2005; Vazdarjanova et al., 2006). For consistency across groups, parameters on the microscope were kept the same for each slide. To date, a detailed stereological analysis of the morphology of the hippocampus and it subfields in GK rats relative to Wistars has not been conducted. While it is true that a change in morphology of the DG may affect a number of physiological measurements in these animals, including gene expression, all data presented here are expressed as proportions of cells expressing Egr1 relative to the total proportion of cells imaged, and any cell loss should affect both the numerator and denominator of this proportion equally.

Expression of *Egr1* was then quantified within the 20% median planes of each image stack. The GCs contained in this volume were classified as either (1) *Egr1* negative, (2) *Egr1* positive within the nucleus, (3) *Egr1* positive within the cytoplasm, or (4) *Egr1* positive within both the nucleus and the cytoplasm (Fig. 1a). Based on the known timing between behavioural stimulation and the expression of *Egr1,* these profiles can be used to provide a histological record of activity using catFISH (Guzowksi et al., 1999; Marrone et al., 2011; Satvat et al., 2011; Gheidi et al., 2013). That is, these gene expression profiles were used to calculate the number of active GCs (as a proportion of the total number of GCs) active during each of 2 distinct behavioural epochs. In the case of the CMT, these epochs correspond to the second sample trial and the test trial. In the environmental exploration, these epochs corresponded to the first and second exploration session.

### 2.6 Statistical analysis

The preference of animals during the first 2 min of the test trial in CMT was analyzed initially by quantifying the time spent exploring each object using established criteria (e.g., Marrone et al., 2018). Briefly, a rat was considered to be exploring an object if its head was oriented towards a part of the object, its nose was within 2 cm of the object, and it was not visibly grooming. If a rat climbed onto an object this was also considered exploration. These exploration times were then used to generate two discrimination ratios (DRs): one for object identity and one for object location. The object identity DR was calculated as:

((OD + OS) – (RD + RS))/(OD + OS + RD + RS)

While the object location DR was calculated as:

((OD + RD) – (OS + RS))/(OD + OS + RD + RS)

A one-way analysis of variance (ANOVA) was used to compare the DRs of GK rats relative to Wistars. In addition, the DRs for each group of rats were compared to zero (chance performance) using a single-sample *t*-test. The transcription of *Egr1* was analyzed by quantifying the total number of GCs transcribing *Egr1* during Epoch 1 and 2 individually, as well as quantifying the probability that the same GC expressed *Egr1* during both behavioural epochs by calculating a similarity score as previously described (Vazdarjanova and Guzowski, 2004). Briefly, the similarity score is derived as follows:

Similarity score = diff(E1E2)/(L - p(E1E2)

Where: (1) Epoch 1 active cells = cytoplasmic Egr1+ *cells*/total cells. (2) Epoch 2 active cells = intra-nuclear Egr1+ cells/total cells. (3) p(E1E2) = Epoch 1 active cells × epoch 2 active cells. This is the probability of cells being active in both epochs, assuming statistically independent neuronal ensembles. (4) diff(E1E2) = (*fraction of cells with Egr1 in both the nucleus and cytoplasm*) - p(E1E2). This provides a measure of deviation from the independence hypothesis. (5) L = the smaller of the ensembles activated by epoch 1 or epoch 2. This normalizes the diff(E1E2) fraction such that complete overlap provides a similarity score of 1 while an amount of overlap equivalent to random chance with replacement provides a similarity score of 0. As an example, consider a scenario in which 10% of the GC population transcribes *Egr1* during each epoch. If 10% of the GCs transcribing *Egr1* during Epoch 2 (i.e., expressing *Egr1* within the nucleus) are the same GCs that transcribed *Egr1* during Epoch 1 (i.e., expressing cytoplasmic *Egr1*), the similarity score becomes: 0.01/(0.1–0.01) = 0.10. However, under conditions in which 90% of the GCs expressing *Egr1* during Epoch 2 are the same GCs that expressed *Egr1* during Epoch 1, the similarity score becomes: 0.09/(0.1–0.01) = 0.91.

All scoring of videos and quantitation of confocal images was done by researchers blind to the experimental conditions. All three of these measures were analyzed using a 2 (strain – GK vs Wistar) by 2 (condition: either CMT vs caged, or same environment vs different environments) ANOVA.

## 3 Results

### 3.1 GK rats show deficits in conjunctive memory recall

**Fig. 3:**
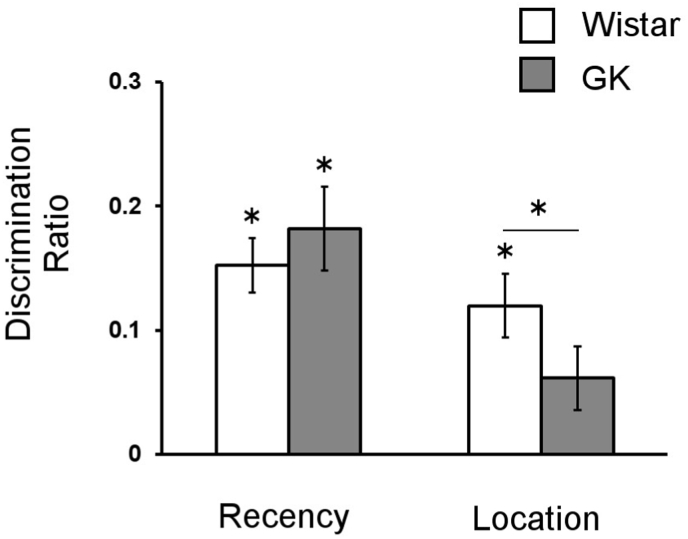
Impaired object-place memory in GK rats. Data show that both Goto-Kakizaki (GK) and Wistars can discriminate objects on the basis of recency, as both groups show better than chance discrimination and group DRs did not differ significantly. The strains differed in their ability to discriminate objects on the basis of location, with only Wistars performing significantly better than chance (data are presented as mean ± SEM, asterisks (*) above individual bars indicate a significant difference from zero, while asterisks over bars indicate a significant difference between strains).

Analysis of the CMT showed that on average, GKs (133 ± 16 s) explored objects for significantly less time than Wistars (184 ± 18 s) during the sample trials (F_1,16_ = 4.50; p = 0.04). This difference in exploration time, however, did not prevent all animals from demonstrating memory for object recency (i.e., “old” vs. “new”). Both groups of animals generated recency DRs that did not differ significantly from each other (F_1,16_ = 0.50; p = 0.49) and both were significantly different from 0 (GK: t_9_ = 5.43, p < 0.001; Wistar: t_7_ = 5.82; p < 0.001). In contrast, a significant difference was apparent between breeds in their ability to discriminate object location (F_1,16_ = 9.20; p = 0.01) and in post-hoc testing, the DRs of Wistar rats was significantly different from zero (t_7_ = 4.23, p < 0.01), while the DRs of GK rats was not (t_9_ = 1.94, p = 0.08). These data (Fig. 3) show that while GK rats remain able to recognize objects, they have a selective deficit in their ability to discriminate changes in object location.

### 3.2 GK rats show altered *Egr1* expression during conjunctive memory recall

**Fig. 4:**
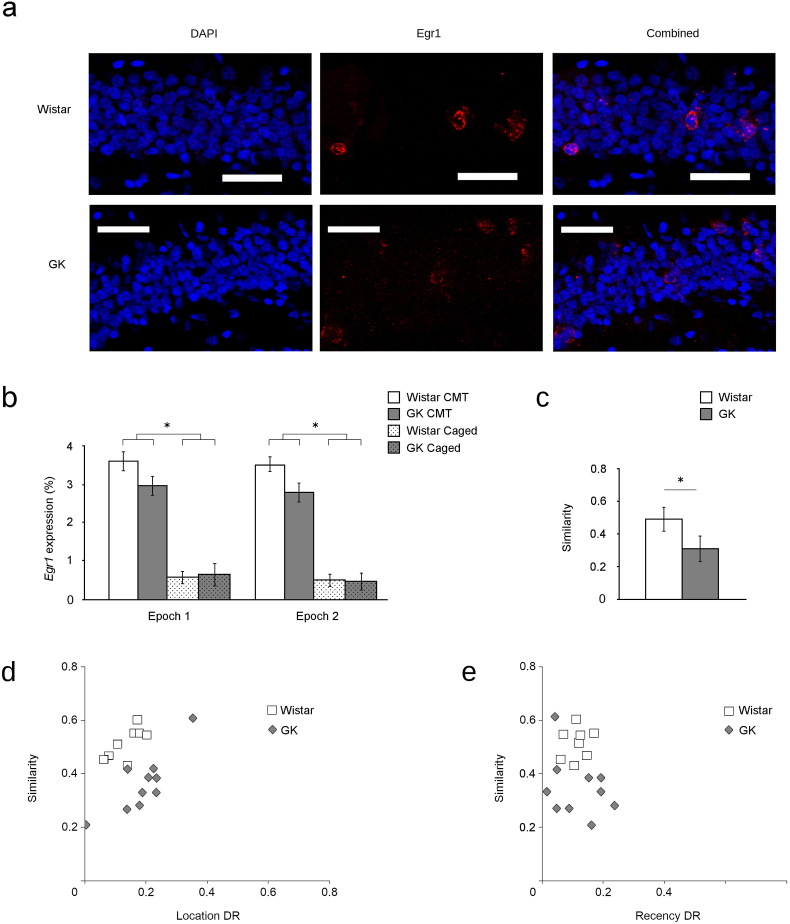
Altered Egr1 expression in GK rats during performance of a conjunctive memory task. Sample confocal micrographs (a, scale bar = 50 μm) of GCs stained with DAPI (blue, left) show that completion of the conjunctive memory task (CMT) results in Goto-Kakizaki (GK) rats having fewer GCs transcribing *Egr1* (red, middle) both within the nucleus and in the surrounding cytoplasm of the same cell, relative to Wistars. Quantifying these observations (b) shows that although both Wistar (white) and GK (grey) rats that completed the CMT transcribe *Egr1* in more GCs relative to caged Wistars (dotted white) or caged GKs (dotted grey), this transcription remains significantly less in GKs that completed the CMT than Wistars. Moreover, a significant difference is observed in the probability that the same GC will transcribe *Egr1* (c). That is, fewer of the GCs that transcribe *Egr1* during the sample trial transcribed *Egr1* again during test. In addition, rats' ability to discriminate object location (d) but not their ability to discriminate object recency (e) is significantly correlated with this decrease in the consistency of *Egr1* transcription [data are mean ± SEM, asterisk (*) = significant difference between groups]. (For interpretation of the references to colour in this figure legend, the reader is referred to the Web version of this article.)

As performance on comparable tasks has been shown to depend on the integrity of the DG (Vazdarjanova and Guzowski, 2004; Vazdarjanova et al., 2006), transcription of *Egr1* was quantified in GCs during this task (Fig. 4a). A significant main effect of condition was observed for *Egr1* transcription both during Epoch 1 (i.e., sample trial 2: F_1,26_ = 9.74; p < 0.01) and during Epoch 2 (i.e., test trial: F_1,26_ = 10.23; p < 0.01), showing that all rats that engaged in the CMT expressed *Egr1* in significantly more granule cells than did caged controls (Fig. 4b). In addition, GKs transcribed *Egr1* in significantly fewer GCs than Wistars (main effect of strain: F_1,26_ = 4.45; p = 0.04). No significant interactions were present.

Analysis of the probability that the **same GCs** transcribe *Egr1* during both trials revealed a significant strain difference (F_1,26_ = 6.92; p = 0.01, Fig. 4c). Thus, in addition to a decrease in the total number of GCs transcribing *Egr1* during each retrieval trial, the GK rats recruit more distinct GC populations to become active in both environments.

In addition, this decrease in the fidelity of *Egr1* transcription correlates with CMT performance. The number of GCs in individual rats that repeatedly transcribed *Egr1* during both behavioural epochs was significantly correlated with individual performance in the CMT (r = 0.491; p < 0.05), consistent with the functional relationship proposed between altered GC activity patterns and memory deficits under high-interference conditions.

### 3.3 GK rats show reduced and less consistent *Egr1* expression during spatial navigation

**Fig. 5:**
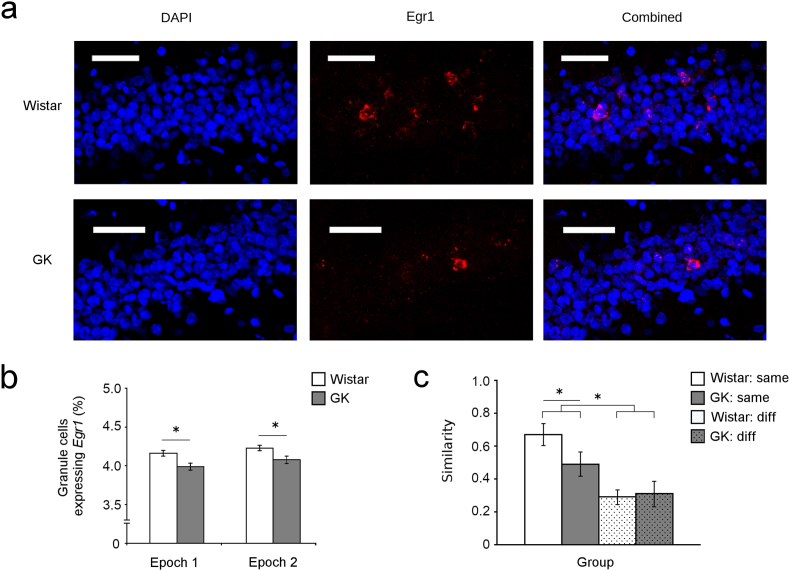
Altered Egr1 expression in GK rats during spatial exploration. Sample confocal micrographs (a, scale bar = 50 μm) of GCs stained with DAPI (blue, left) from animals exploring 2 distinct novel environments shows that under these conditions, Goto-Kakizaki (GK) rats having fewer GCs transcribing *Egr1* (red, middle) both within the nucleus and in the surrounding cytoplasm of the same cell, relative to Wistars. Analysis of *Egr1* transcription (b) shows transcription of *Egr1* in ∼4% of the total putative GC population examined, and no significant effect of condition (i.e., same vs. different) was observed on the number of GCs transcribing *Egr1*. Relative to Wistar rats (white), GKs (grey) show a small but significant decrease in the number of GCs transcribing *Egr1*. A significant difference is also observed in the probability that the same GC will transcribe *Egr1* (c). Animals that visited the same environment twice were far more likely to transcribe *Egr1* in the same GCs on both occasions compared to rats this explored 2 distinct spaces. No difference in similarity scores can be observed in animals that visit 2 different environments. In animals that visited the same environment twice, GKs had fewer GCs that consistently transcribe *Egr1* during both visits. This difference is not apparent in animals that visited 2 different (diff) environments. Results from these controlled conditions confirm the observations made in animals engaging in CMT, showing that GCs transcribe *Egr1* less consistently in GK animals even under more controlled conditions [data are mean ± SEM, asterisk (*) = significant difference between groups]. (For interpretation of the references to colour in this figure legend, the reader is referred to the Web version of this article.)

In any spontaneous recognition task that relies on animals demonstrating recognition by dwelling in the proximity of an object, performance is confounded with differences in the amount of space occupied by an animal, particularly given observations that GK rats have reduced spontaneous exploration (Moreira et al., 2007b). Because the firing of place cells depend on an animal travelling through that cell's receptive place field, and animals travelling through more space necessarily elicit the firing of more place cells, changes in occupancy of space can drive changes in place-cell activity and thus immediate-early gene expression (Nakamura et al., 2010). This fact has lead researchers to devise some means to control for immediate-early gene expression, either by controlling the movement of animals (e.g., Guzowski et al., 1999; Marrone et al., 2011), or devising a means to use spatial occupancy to normalize gene expression data (e.g., Hartzell et al., 2013). Here we use assisted spatial exploration (Fig. 5) to control spatial occupancy between animals. Results from these controlled conditions confirm the observations made in animals engaging in CMT. No significant effect of condition (i.e., same vs. different) was observed on the number of GCs transcribing *Egr1* (Fig. 5b) in either epoch 1 (F_1,28_ = 1.31; p = 0.26) or epoch 2 (F_1,28_ = 1.46; p = 0.24), consistent with previous reports (Chawla et al., 2005; Satvat et al., 2011). A significant effect of strain (F_1,28_ = 4.93; p = 0.03) shows that, consistent with observations made in animals completing the CMT, fewer GCs transcribed *Egr1* in GK animals.

Analysis of similarity scores shows a significant effect of condition (F_1,28_ = 7.08; p = 0.01) showing that animals that visited the same environment twice were far more likely to transcribed *Egr1* in the same GCs on both occasions compared to rats this explored 2 distinct spaces. This pattern is consistent with both gene expression (e.g., Marrone et al., 2011) and physiological recordings (e.g., Goodsmith et al., 2017) showing that the same GCs are reliably recruited to express place fields across multiple episodes of experience in the same environment and different GCs are recruited in distinct environments. No significant main effect of strain was present (F_1,28_ = 2.76; p = 0.11), but a significant interaction was observed (F_1,28_ = 5.09; p = 0.03). This interaction shows that in GK rats fewer GCs transcribed *Egr1* repeatedly only when they visited the same environment repeatedly. No difference in similarity scores can be observed in animals that visit 2 different environments. These data show that when rats are exposed to the same environment on 2 distinct occasions, the GCs of GK rats are less likely to transcribe *Egr1* in the same GCs during both exposures. This pattern suggests that the place representations in GKs are less stable (i.e., are more likely to remap) than the GCs of Wistars during repeated exposure to similar stimuli. When highly distinct local and distal cues are present (i.e., in 2 different environments with unique local and distal cues), GCs in GK and Wistar rats are equally likely to transcribe *Egr1* during both exploration sessions.

## 4 Discussion

The current data show that within 3 months of CHAIR, GK rats show a selective deficit in binding the memory of an object with its precise location. Since the ability for rodents to complete similar tasks depends on the integrity of the DG (Dees and Kesner, 2013; Kesner et al., 2015), the behavioural data reported here strongly suggest aberrant activity in GCs. This hypothesis fits well with observations that (a) the probability that the same GCs will consistently transcribe *Egr1* in response to two exposures to similar (or even identical) stimuli is significantly lower in GKs, and (b) this inconsistency predicts memory impairment across individuals. Collectively, these data suggest that the DG is a major site of brain dysfunction in response to CHAIR.

Two distinct phenomena could contribute to changes in *Egr1* expression in GK rats. Changes may result from a difference in the underlying pattern of neuronal activity that is driving *Egr1* expression, or from a change in electrotranscriptional coupling (Guzowski et al., 2006) – the relationship between *Egr1* expression and the underlying activity. While changes in electrotranscriptional coupling cannot be ruled out entirely, the observation that *Egr1* expression is equivalent across strains in caged controls as well as in rats exploring 2 novel environments strongly suggests that CHAIR does not change *Egr1* expression *per se*, but rather the information processing in perforant path neural circuits. In particular, the environmental exploration group that was exposed to 2 distinct environments has an equivalent experience. They spend the same amount of time ambulating in 2 sessions with the same timing, but in the presence of visually distinct cues, and thus presumably without the need for pattern separation. In this condition, the pattern of activity in GK and Wistar rats is equivalent, consistent with the idea that this difference in activity patterns in GCs as GK rats discriminate object displacement emerge because of differences in pattern separation-related computation in the DG of GK rats. These proposed changes in information processing in GK rats are consistent with human imaging data showing that performance in a comparable object recognition task is exquisitely sensitive to perforant path functional integrity (Bakker et al., 2008; Yassa et al., 2010) and the DG in particular (Baker et al., 2016).

These observations do not preclude the possibility that CHAIR affects other neural systems that are engaged by this task. In particular, the prefrontal (e.g., Barker et al., 2007; Cross et al., 2012), perirhinal (e.g., Bussey et al., 2001; Liu and Bilkey, 2001), and retrosplenial (e.g., Powell et al., 2017; Hayashi et al., 2020) cortices all contribute to performance in this task, although lesions to all of these regions generally also impair discriminations based on recency (e.g., Bussey et al., 1999; Liu and Bilkey, 2001). In contrast, lesions to the DG are sufficient to create deficits in detecting object displacement and not recency (Gilbert et al., 2001), and comparable impairments can be created solely by reducing the integrity of the perforant path (Burke et al., 2018). It should be noted, however, that selective lesions to CA1 also produce this pattern of deficits (Hunsaker et al., 2008).

It is intriguing that several animal models of age-related disease demonstrate deficits in object location recognition at earlier ages, and later show object recognition deficits (e.g., Billings et al., 2005; Gulinello et al., 2009). Thus, it is possible that more robust deficits will progressively emerge in GK rats as they spend more time in CHAIR. However, it may also be that the task is not ideally designed to capture PrC deficits, as results obtained in object recognition tasks are highly sensitive to the exact behavioral protocol used (Marrone et al., 2018). Both of these factors may explain why the current data are at odds with two previous reports of object recognition deficits in GK rats (Prakash et al., 2013; Hardigan et al., 2016).

It should be noted, however, that a number of alternative explanations may be provided for the results obtained here. Notably, GKs exhibit decreased motivation including decreased exploration of novelty (Moreira et al., 2007b). Consistent with these data, GKs spent less total time exploring the objects during the sample phases. Presumably, the binding of an object with its context is more difficult than remembering only an object's identity. Based on this assumption, it is possible that the conjunctive memory requires more exploration time to form. As such, GK rats may explore enough to form object memories, but not object-location conjunctions, making their recognition memory deficits at least in part the result of differences in motivation to explore novelty. In addition, reduced visual acuity may play a role in the observed deficits. A number of studies have shown retinopathy in GK rats (e.g., Miyamoto et al., 1996; Allen et al., 2019) that mimics the pathology seen in humans with T2DM (Kowalczuk et al., 2013). It is possible that decreased visual acuity leads to GK rats being less able to see distal cues. In this hypothetical case, recency discriminations could be successfully made based solely on local visual information (potentially with the aid of tactile information), but discriminating object displacement, which requires binding local visual features of an object with the distal visual scene, may be impaired. However, the observation that GK rats can find a hidden platform in the Morris water maze at a rate greater than chance (e.g., Xiang et al., 2015; Li et al., 2013, 2017; Tian et al., 2018; Ke et al., 2020) indicates that they can, in fact, see distal cues *to some degree.* In addition, GK rats show deficits in 2-choice positional discrimination operant task (Moreira et al., 2007a) that recruits the hippocampus (Roman and Soumireu-Mourat, 1988; Sif et al., 1991) and depends solely on local cues. Thus, the balance of evidence suggests that distal visual acuity cannot account for all of the spatial learning deficits of GK rats.

The pattern of these deficits are consistent with the hypothesis that T2DM accelerates the emergence of a senescent phenotype in the medial temporal lobe. In fact, the similarity between the changes in both memory and hippocampal function observed here and those phenotypically seen in aged rodents (Small et al., 2011; Lester et al., 2017) is striking. At 4 months of age, GK rats develop deficits in a high-interference memory task comparable to much older rodents (Johnson et al., 2016; Marrone et al., 2018). In addition, the decrease in *Egr1* expression is consistent with observations of aged GCs (Small et al., 2004; Marrone et al., 2012a, 2012b; Gheidi et al., 2013). In particular, the decrease in the fidelity of GC *Egr1* expression following spatial exploration in similar contexts is also observed in aged rats (Marrone et al., 2011). This remarkable consistency between changes to both DG-dependent behaviour and GC gene expression suggests that CHAIR induces similar changes to information processing in the medial temporal lobe architecture as normal aging.

Progressive age is known to lead to several major changes in the architecture of the DG that have the potential to alter GC recruitment and result in memory impairment. Aging is accompanied by a substantial reduction in the entorhinal innervation to the DG (Geinisman et al., 1992). This decrease in input is the most likely cause for the general decrease in the number of GCs activated during a single bout of spatial exploration in aged rats and is likely to contribute to memory deficits in high-interference conditions. Consistent with this hypothesis, partial transection of the perforant path is sufficient to induce memory deficits selectively in high-interference conditions (Burke et al., 2018). It is likely that GK rats have a similar reduction in perforant path input given observations of reduced excitatory postsynaptic markers (Matsunaga et al., 2016). This observation, combined with the fact that imaging studies show that mnemonic discrimination impairments are associated with reduced integrity of white matter afferents to the hippocampus (Bennett and Stark, 2016; Yassa et al., 2010) provide further evidence for a convergent mechanism of impaired memory related to both CHAIR and normal aging.

Taken together, these data suggests that CHAIR functions to accelerate age-related changes in medial temporal lobe architecture and confer a senescence phenotype on the hippocampus. This suggests a common etiology for age- and diabetes-related cognitive impairment and, importantly, suggests that interventions that can ameliorate age-related cognitive decline would also benefit individuals with T2DM at any age.

## CRediT authorship contribution statement

**Chelsey C. Damphousse:** Conceptualization, Methodology, Investigation, Data curation, Visualization, Formal analysis, Writing – original draft. **Jaclyn K. Medeiros:** Investigation, Writing – original draft. **Nicole E. Micks:** Investigation. **Diano F. Marrone:** Conceptualization, Methodology, Visualization, Writing – review & editing, Supervision, Funding acquisition.

## Declaration of competing interest

The authors declare that they have no known competing financial interests or personal relationships that could have appeared to influence the work reported in this paper.

## Data Availability

Data will be made available on request.
